# *Streptococcus suis*: A Review of Its Effects on Immune Organs

**DOI:** 10.3390/microorganisms13071613

**Published:** 2025-07-09

**Authors:** Siyu Pan, Haijuan He, Tong-Qing An, Shujie Wang

**Affiliations:** 1 National Key Laboratory of Veterinary Biotechnology, Harbin Veterinary Research Institute, Chinese Academy of Agricultural Sciences, Harbin 150001, China; 2 Institute of Animal Husbandry, Heilongjiang Academy of Agriculture Sciences, Harbin 150086, China; he_haijuan@haas.cn; 3 Heilongjiang Provincial Key Laboratory of Veterinary Immunology, Harbin 150069, China

**Keywords:** *Streptococcus suis*, immunosuppressive, thymus, spleen, lymph nodes

## Abstract

*Streptococcus suis* (*S. suis*) is a major pathogen in pigs and an emerging zoonotic agent which causes serious infections in humans. It is also an immunosuppressive pathogen that exerts detrimental effects on the thymus, spleen, lymph nodes, and macrophages, impairing their ability to perform their normal physiological functions. *S. suis* induces thymic atrophy, splenomegaly, and lymphadenectasis and triggers apoptosis in T cells and B cells, as well as pyroptosis in macrophages within immune organs. Subsequently, T cell subsets in peripheral blood become abnormal, and the expression of cytokines becomes dysregulated, which leads to host immunosuppression, suggesting a new virulence mechanism of *S. suis*.

## 1. Introduction

*Streptococcus suis* (*S. suis*) is a major porcine pathogen responsible for significant economic losses in the global swine industry [1]. It is one of the leading causes of bacterial mortality in postweaned piglets aged 5 to 10 weeks and is also recognized as an emerging zoonotic pathogen [1]. To date, 29 serotypes have been identified based on capsular polysaccharide (CPS) antigens, with serotype 2 being the most prevalent and virulent [2]. *S. suis* initially colonizes the host and subsequently evades the host immune response, enabling the persistence and dissemination of the infection [3].

*S. suis* can disrupt both the innate and adaptive immune systems by targeting key immune organs, cells, and cytokine networks [4]. Recent studies demonstrate that *S. suis* induces damage to immune organs such as the thymus, spleen, and lymph nodes [5,6,7]. This leads to imbalanced cytokine production favoring the release of pro-inflammatory cytokines, which exacerbates tissue damage and prolongs infection [8,9,10], Additionally, T cell responses are disrupted, with *S. suis* modulating their activation and memory response, rendering the immune response ineffective [11].

Despite extensive research on *S. suis* pathogenesis and its interaction with individual immune components, a comprehensive synthesis focusing specifically on its systemic impact on the architecture and function of major immune organs is lacking. The precise mechanisms by which *S. suis* disrupts the delicate interplay within and between these organs (e.g., thymus, spleen, lymph nodes), leading to the observed global immune dysregulation and ineffective clearance, remain incompletely understood. Furthermore, the relative contribution of damage to specific immune organs to the overall pathogenesis and persistence of *S. suis* infection requires further elucidation. This review therefore aims to summarize and critically evaluate the current knowledge regarding the damage inflicted by *S. suis* on immune organs and the mechanisms underlying *S. suis*-induced immune dysregulation. Specifically, it seeks to integrate findings across studies to provide a holistic view of how *S. suis* subverts the host defense by targeting these vital immune structures, thereby addressing the identified knowledge gaps and highlighting avenues for future research aimed at developing more effective interventions.

## 2. The Thymus and *S. suis*

The thymus is essential for T cell maturation and immune tolerance [12,13]. Structurally, the thymus consists of two main regions: the cortex and the medulla [14]. The cortex is densely populated with immature thymocytes and serves as the site of positive selection [15], which recognize self-major histocompatibility complex (MHC) molecules for further development [16]. In contrast, the medulla contains more mature thymocytes and serves as the site of negative selection, which eliminates self-reactive T cells that could cause autoimmunity [17]. At birth, the thymus consists of two lobes, covered by a connective tissue capsule, and plays a critical role in T cell development [13,18]. Postnatally, T cell precursors, known as prothymocytes, are generated in the bone marrow and subsequently migrate to the thymic cortex, where they undergo further maturation and selection [19].

**Figure 1:**
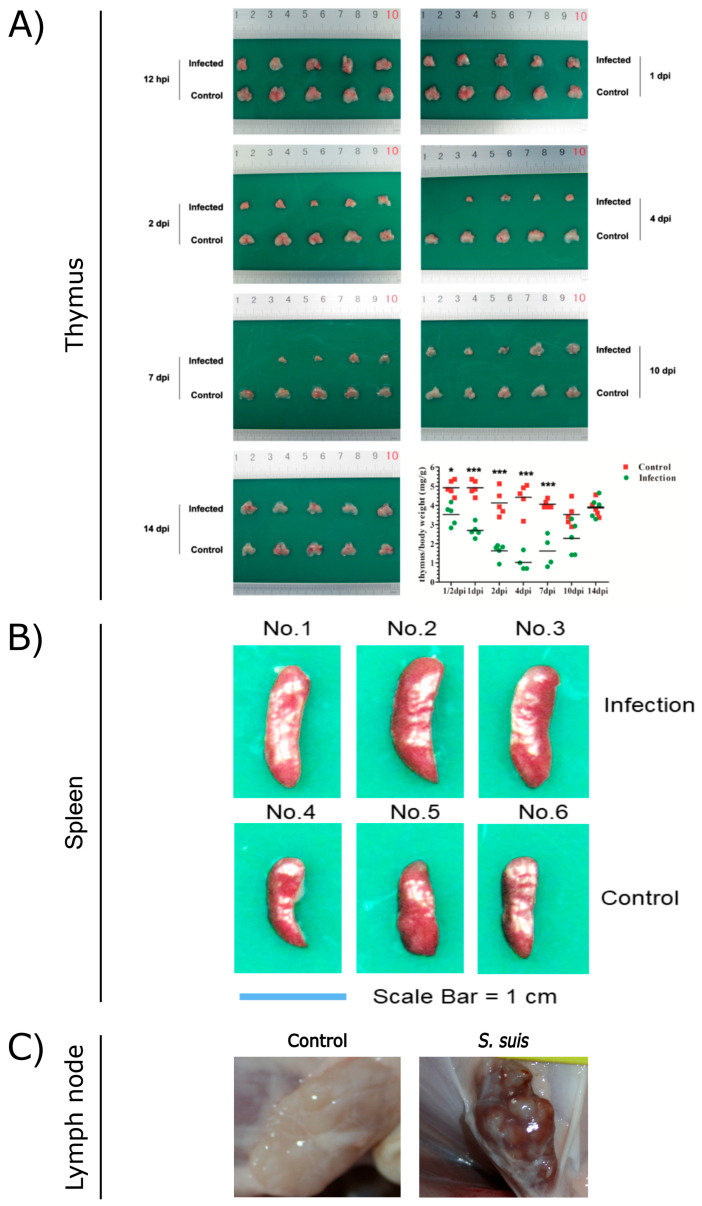
Pathological observations of thymus (**A**), spleen (**B**), and lymph nodes (**C**) from *S. suis*-infected and control pigs. (**A**) Reproduced from Reference [6] (https://doi.org/10.1128/iai.00950-19), *, *p* < 0.05; ***, *p* < 0.001; (**B**) reproduced from Reference [5] (https://doi.org/10.1128/spectrum.03210-22); (**C**) reproduced from Reference [7] (https://doi.org/10.3389/fmicb.2023.1159590).

As a critical primary lymphoid organ, the thymus plays a key role in the immune response. However, *S. suis* can induce severe damage to the thymus of mice. Macroscopically, the thymus of infected mice exhibited progressive atrophy, with a noticeable reduction in size starting from 1 day post-infection (dpi). By 2 dpi, the thymus size was reduced by 50–80% compared to the control group. From 4 to 7 dpi, infected mice displayed severe thymic atrophy, losing normal morphology (Figure 1A). Histopathologically, the thymic lobules of infected mice showed a marked decrease in size, accompanied by a significant reduction in lymphocyte numbers and disintegration/necrosis of the cortical boundary. The medulla appeared blurred, with signs of atrophy or disappearance during the 1–7 dpi period. Similarly, *S. suis* infection in swine induced thymic atrophy, as evidenced by thymus/body weight ratios of 0.464 and 0.73 g/kg at 5 dpi, which were significantly lower than those of control piglets. Collectively, these findings indicate that thymic atrophy occurs in both *S. suis*-infected mice and piglets.

*S. suis* infection induces severe thymic atrophy, consistent with the pathological lesions observed in other immunosuppressive pathogens such as porcine circovirus type 2 (PCV2) [20,21,22] and highly Porcine Reproductive and Respiratory Syndrome Virus (HP-PRRSV) [23,24,25]. In the atrophied thymus infected with PCV2, the imbalance of cytokine mRNA, especially the increase in the IL-10 mRNA level, combined with the abnormalities in histopathology and blood routine, highly indicated T cell immunosuppression and that the immune system of pigs was severely impaired [21]. In the HP-PRRSV-infected pigs, the thymus showed 50–90% reductions in size in comparison with a normal thymus [24]. The thymus was reduced in size on 7 dpi, with thymus weight/body weight ratios of 0.433, and continued to shrink on 14 dpi with thymus weight/body weight ratios of 0.144 [25]. Histopathologically, with the increase in infection time, the size of the thymus lobules decreased, the boundary between the thymus cortex and medulla became blurred, and cortical atrophy became more severe.

There are numerous serotypes of *S. suis*, but they do not wholly determine the pathogen’s virulence. *S. suis* isolates exhibit varying capacities to induce thymic atrophy depending on their virulence levels. Highly virulent isolates, such as 700794 (serotype 2) and BM0806 (serotype 7), caused severe thymic atrophy in murine models, with thymus/body weight ratios of 1.156 and 0.957 mg/g at 4 dpi, respectively. Moderately virulent isolates, including HG1210 (serotype 9) and M1302 (serotype 7), induced mild thymic atrophy, with thymus/body weight ratios of 3.498 and 2.288 mg/g at 4 dpi, respectively. In contrast, the nonvirulent isolate W7119 (serotype 9) did not cause any observable thymic atrophy [6]. Virulence-dependent thymic atrophy in *S. suis* infection reflects broader immunosuppressive patterns linked to pathogenicity. As illustrated, during PRRSV infection, the highly virulent strains Lena and HuN4 induced severe thymic atrophy [25], whereas the low-virulence strains 3249 and CH-1a caused minimal lesions [26,27].

In the *S. suis*-infected thymus, the infection disrupts mitochondrial membrane permeability in thymic cells, accompanied by increased expression of the Bax protein and decreased Bcl2 levels [6]. This process may be mediated by bacterial virulence factors such as suilysin, which disrupts mitochondrial membrane potential and activates the intrinsic apoptotic pathway [28]. Increased mitochondrial membrane permeability triggers the release of cytochrome C (CytC) and apoptosis-inducing factor (AIF) into the cytoplasm [29]. The mitochondrial AIF triggers chromatin condensation and DNA fragmentation and allows nuclei to undergo apoptosis [30]. Thus, the P53 signaling pathway is involved in apoptosis of thymic cells induced by *S. suis*. Meanwhile, *S. suis* infection also activates the caspase cascade in thymic cells, shown by cleaved caspase-8, -9, and -3 fragments, which shows that the caspase pathway is also involved in apoptosis induced by *S. suis*. Apoptosis reduces the number of CD3^+^CD4^+^CD8^+^ thymic cells, and consequently, the number of CD4^+^CD8^−^ and CD4^−^CD8^+^ T cells in peripheral blood decreases significantly. In addition, significant changes in the cytokine profile occur, especially the dysregulation of pro-inflammatory cytokines such as IL-2, IL-4, IL-6, IL-10, IFN-β, and TNF-α, which influence host immune responses [6]. Altogether, thymic atrophy is caused by the depletion of thymic cells, which disrupts immune homeostasis, helping pathogens avoid immune clearance. In HP-PRRSV-infected piglets, there were many apoptotic cells in the thymus on 3 dpi, and the number of apoptotic cells increased significantly on 7 dpi and 10 dpi according to histological sections and electron micrograph observations [25]. Moreover, there was an increase in expressing molecules related to the extrinsic pathway of apoptosis (cCasp8 and Fas), indicating an important role in inducing thymocyte apoptosis in HP-PRRSV-infected piglets [26,31]. The thymus underwent extensive apoptosis, resulting in a reduction in the number of mature T lymphocytes and the continuous release of viral particles, which may explain why the clinical symptoms of pigs infected with HP-PRRSV were more severe [16,26]. In addition, HP-PRRSV attacks CD80- and calgranulin + calcium-positive cells (such as dendritic cells (DCs) and macrophages) in thymic tissue and causes a reduction in visible cellular components, which also play an important role in the pathogenesis of thymic atrophy [32].

## 3. The Spleen and *S. suis*

The spleen is a vital immune organ in mammals [33]. Structurally, it is divided into two primary regions, red pulp and white pulp, which are delineated by the marginal zone [34]. The red pulp primarily functions in blood filtration by eliminating senescent red blood cells, recycling iron, and harboring macrophages essential for pathogen clearance [35,36]. The white pulp, enriched with T and B cells, serves as the site where adaptive immune responses are initiated. Specifically, T cells are predominantly located in the periarteriolar lymphoid sheath, while B cells reside within the follicles [37,38]. The marginal zone plays a pivotal role in capturing and processing pathogens, facilitating efficient antigen presentation and T cell activation [39]. Beyond filtering functions, the spleen maintains immune tolerance, prevents autoimmunity, and modulates immune responses [40,41]. It houses various immune cells, including natural killer (NK) cells and natural killer T (NKT) cells [42]. Therefore, the spleen is critical for maintaining immune homeostasis, defending against infections, and preventing excessive immune reactions.

*S. suis* infection induces inflammatory splenomegaly. Wang et al. utilized C57BL/6 mice to replicate the spleen lesions observed in *S. suis*-infected piglets. The spleens of infected mice began to exhibit enlargement from 2 dpi, with a significantly increased spleen/body weight ratio of 5.346 (*p* < 0.05). This enlargement persisted relative to control spleens, with further increases noted at 4 dpi and 7 dpi (Figure 1B). Histopathologically, a marked reduction in lymphocyte numbers within the white pulp was observed, accompanied by splenocyte depletion leading to focal lesions in the white pulp at 1–4 dpi in *S. suis*-infected mice [5]. HP-PRRSV can also cause splenomegaly in pigs, but the difference is spleen swelling with scattered infarction or white spots on the surface [24]. Similarly, splenomegaly is one of the characteristic symptoms of African swine fever virus (ASFV) infection [43]. But the typical pathological feature of classical swine fever virus (CSFV) infection is splenic infarction with a decrease in splenic lymphocytes [44].

The study conducted by Wang et al. revealed that pro-inflammatory cytokines, including IL-6, IFN-β, and TNF-α, exhibited a marked increase in infected mice spleens and reached their peak at 1 dpi before gradually declining [5]. Similarly, Li et al. demonstrated that the expression of genes associated with cytokines and inflammatory pathways significantly increased in infected spleens [45]. Concurrently, the expression of toll-like receptor 2 (TLR2) was upregulated at 1 dpi, whereas TLR4 expression remained unchanged [5]. Unlike *S. suis* infection, CSFV infection leads to the downregulation of TLR2 and TLR4 expression in the spleen, while TLR3 and TLR7 expression increase [44]. Transcriptional analysis further indicated that the initial immune recognition of highly pathogenic *S. suis* serotype 2 was mediated by the TLR2 pathway, subsequently triggering a cascade of pro-inflammatory cytokines [45]. Moreover, the expression of genes involved in transcriptional regulation, cellular transport, and metabolism decreased significantly, reflecting the inhibition of normal cellular activity in infected splenocytes. *S. suis* evades phagocytosis, enabling continuous colonization and toxin production within spleen tissue, which exacerbates tissue damage and perpetuates immune dysregulation [45].

The study conducted by Wang et al. also revealed that *S. suis* triggered apoptosis in B cells of infected spleens of mice [5]. In the spleens of infected mice, caspase-8 was upregulated and there was no significant change in caspase-9 and CytC, indicating that the extrinsic caspase pathway contributed to *S. suis*-induced splenocytes apoptosis. H. Gou et al. demonstrated that CSFV infection induced apoptosis in the spleen, mechanistically linked to lymphocyte depletion. Western blot revealed that the level of cleaved caspase-3 in CSFV-infected splenocytes increased, accompanied by concurrent activation of both caspase-8 and caspase-9, which indicated synergistic engagement of extrinsic and intrinsic apoptosis pathways [46]. *S. suis* could induce the cleavage of pro-caspase-1 and gasdermin D (GSDMD) and increase the expression of IL-1β and IL-18 in spleens of infected mice, which suggests that *S. suis* caused pyroptosis in splenocytes [5]. Pyroptosis occurs via the NOD-like Receptor Pyrin domain-containing 3 (NLRP3) inflammasome pathway, involving the activation of caspase-1 and the cleavage of GSDMD. Studies have demonstrated that high expression of suilysin in *S. suis* is closely associated with the activation of the NLRP3 inflammasome [47,48,49], and the membrane-perforating activity of suilysin plays a critical role in inducing high levels of NLRP3 inflammasome activation [50]. Thus, suilysin may be an important factor causing splenomegaly in *S. suis*-infected spleens.

Macrophages are polarized to the M1 phenotype upon receiving signals from *S. suis* in the surrounding microenvironment. Both M1 polarization and pyroptosis of macrophages release pro-inflammatory mediators, including IL-1β, IL-6, IL-18, IL-17A, C-X-C Motif Chemokine Ligand 8 (CXCL8), and TNF-α [45], which exacerbate inflammation and tissue damage. Additionally, the c-Jun N-terminal kinase (JNK) and p38-mediated Mitogen-Activated Protein Kinase (MAPK) pathways are involved in pro-inflammatory responses, further amplifying splenic inflammation. Meanwhile, the expression of regulatory T cells (Tregs) significantly increases at 1 dpi, which may suppress T cell proliferation and secrete inhibitory cytokines. Consequently, anti-inflammatory cytokine IL-10 and transforming growth factor beta 1 (TGF-β1) are inhibited during early infection [51]. The release of a large number of inflammatory mediators promotes the extravasation of liquid components from the blood into the interstitial spaces, leading to splenomegaly [5]. *S. suis*-induced splenocytes depletion is alleviated in TNF-α-/- mice in the early stage of infection, which indicates that the inflammatory mediator TNF-α targets B cells and induces apoptosis through the TNF receptor in spleens.

## 4. The Lymph Nodes and *S. suis*

Lymph nodes are secondary lymphoid organs that function as central hubs for immune activation and regulation [52]. Structurally, they are composed of three distinct regions: the cortex, paracortex, and medulla [53]. The cortex houses B cell follicles, where germinal centers facilitate B cell activation and antibody affinity maturation. In contrast, the paracortex is predominantly occupied by T cells and serves as the site for antigen presentation by DCs [54]. The medulla filters lymph through medullary cords and sinuses, where B cells, plasma cells, and macrophages remove pathogens and foreign substances [55]. Lymph nodes serve as filters for lymph fluid, capturing antigens and pathogens to initiate adaptive immune responses. Follicular DCs (FDCs) retain antigens for prolonged stimulation of B cells, thereby supporting the production of high-affinity antibodies [56]. Moreover, lymph node stromal cells contribute to peripheral tolerance by expressing peripheral tissue-restricted antigens (PTAs), which regulate self-reactive T cells and prevent autoimmunity [57].

During infections, lymph nodes coordinate both innate and adaptive immune responses, ensuring efficient pathogen clearance [58]. Different lymph nodes of severely diseased piglets are often infected with the same aggressive strain of *S. suis* and spread throughout the body. The host–pathogen interaction in the lymph nodes is an important part of the pathogenesis of *S. suis*, and the immune escape mechanism of *S. suis* allows the pathogen to survive in the lymph nodes for a longer time [59]. *S. suis* has been shown to attach to lymphocytes, and lymph nodes may contain *S. suis*-positive lymphocytes [59,60]. In piglets infected with *S. suis*, inguinal lymph nodes (ILNs) showed lymphadenectasis or hemorrhagic lesions (Figure 1C) on 14 dpi. Histopathological analysis revealed significant depletion of lymphocytes, indistinct lymphoid follicle architecture, and eosinophil infiltration. *S. suis* infection secondary to PRRSV promotes the depletion of ILNs cells, suggesting that *S. suis* can exacerbate damage to ILNs [7,61]. The study by Carlos et al. demonstrated that the host is capable of rapidly controlling nonvirulent *S. suis* at the inoculation site, mediated through a sustained immune response in the relevant lymph nodes. In contrast, the virulent strains appear to inhibit a robust lymph node response and remain localized at the inoculation site, where they continue to elicit inflammatory mediators [62].

*S. suis* infection induces lymphadenectasis and hemorrhage, with reductions in the number of lymphocytes. Necrosis and apoptosis were observed in lymphocytes within the microscopic lesions of *S. suis*-infected ILNs, which may serve as a direct contributing factor to lymphocytopenia. Furthermore, double immunofluorescence staining with TUNEL showed that *S. suis* mainly induces caspase-dependent apoptosis in ILNs [7]. In addition, the cause of lymphadenopathy and hemorrhage is also related to the inflammatory mediators, which is the same mechanism as in splenomegaly. Similarly, other swine pathogens such as PCV2 [63], PRRSV [24,64], and ASFV can also cause lymph node swelling and hemorrhage. Under ASFV infection, autopsy results showed that multiple lymph nodes had hemorrhaged in various parts of the body and presented a “marble” appearance, and there was severe lymphocyte depletion due to apoptosis [65]. ASFV infection induces lymph node enlargement and severe hemorrhage, which may be due to the fact that ASFV mainly infects monocytes/macrophages during the invasion process. The lymph nodes are the core organs of the innate immune system and are rich in such target cells. Consequently, viral replication is most active and the viral content is the highest in the lymph nodes [66,67]. Unlike *S. suis* infection, which induces lymphocyte necrosis, ASFV infection primarily triggers lymphocyte apoptosis. Apoptosis manifests in T lymphocytes during early infection (4 dpi) and extends to follicular B lymphocytes in later stages. In pigs that recovered back to health after being infected with the sublethal Malta strain, researchers found that the virus remained in the lymph nodes for up to 48 dpi, and within 32 dpi, the virus was present in the paracortex cells of the lymph nodes, surrounded by apoptotic lymphocytes. These observations suggest that apoptosis of uninfected lymphocytes may be induced by cytokines or apoptotic mediators released by macrophages infected with ASFV [68]. During acute ASFV infection, elevated levels of pro-inflammatory cytokines IL-1α, IL-1β, IL-6, and TNF and chemokines CCL2, CCL5, and CXCL10 were detected in the lymph nodes using qPCR methods, which correlate with hemorrhage, edema, and lymphocyte depletion.

## 5. Macrophages and *S. suis*

Macrophages are immune cells that perform a variety of functions, including phagocytosis, cytokines secretion, and antigen presentation, thereby bridging innate and adaptive immunity [69,70]. Their functional plasticity enables them to polarize into the pro-inflammatory M1 phenotype or the anti-inflammatory M2 phenotype in response to environmental stimuli [71,72,73]. M1 macrophages, induced by interferon (IFN)-γ and microbial signals, produce cytokines such as tumor necrosis factor (TNF)-α and interleukin (IL)-6 [74,75]. These cytokines play a crucial role in pathogen clearance, but their overactivation can lead to tissue damage [74,76]. In contrast, IL-4-stimulated M2 macrophages facilitate tissue repair and immunosuppression by releasing anti-inflammatory cytokines like IL-10 [77,78]. Tissue-specific macrophages, such as Kupffer cells in the liver and microglia in the brain, exemplify their adaptability, contributing to homeostasis and specialized functions within their respective environments [79,80,81].

Both virulent and avirulent *S. suis* strains exhibit cytotoxic effects on macrophages, as evidenced by an increase in lactate dehydrogenase (LDH) activity throughout the culture period [41,82]. Furthermore, these strains mediate macrophage polarization, predominantly towards the pro-inflammatory M1 phenotype, characterized by elevated levels of IL-1β as a marker [83]. Similarly, PRRSV infection can also induce macrophages to polarize towards the M1 phenotype. Following PRRSV infection of porcine alveolar macrophages (PAMs), the mRNA levels of M1 polarization-related factors (including IL-6, TNF-α, CD86, and CXCL-10) gradually increased, reaching a peak at 12 h post-infection (hpi) or 24 hpi. Meanwhile, the transcriptional levels of factors related to M2 polarization (including IL-10, Arg-1, and CD206) remained at a relatively low level. Additionally, the protein level of M1 polarization-related factor IL-12 significantly increased in PRRSV-infected PAMs [84].

**Figure 2:**
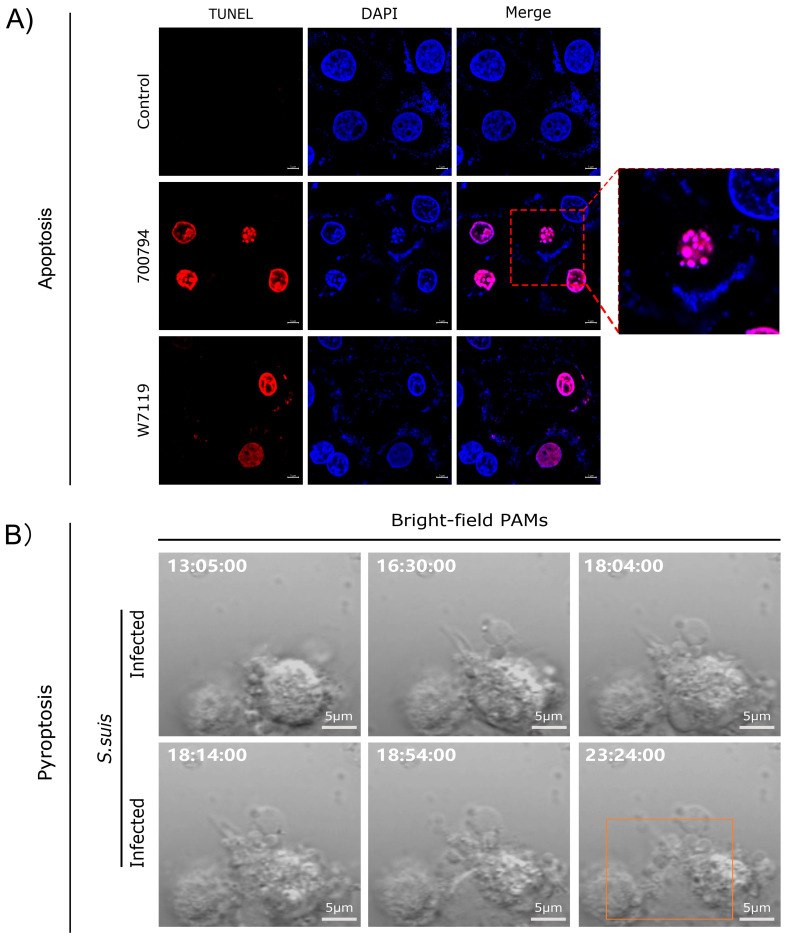
Analysis of apoptosis and pyroptosis in macrophages infected with *S. suis*. (**A**) Reproduced from Reference [85] (https://doi.org/10.3390/microorganisms11010160); (**B**) reproduced from Reference [83] (https://doi.org/10.3390/microorganisms12091879), orange rectangle indicate pyrolyzed cells.

Live-cell imaging using propidium iodide (PI) staining combined with terminal deoxynucleotidyl transferase (TdT)-mediated deoxyuridine triphosphate (dUTP)–biotin nick end-labeling (TUNEL) assays revealed that *S. suis* promoted macrophage apoptosis (Figure 2A), resulting in apoptotic bodies consisting of membrane-bound fragments with condensed cytoplasm and nuclei. Notably, the number of apoptotic cells induced by the virulent strain 700794 was significantly higher (*p* < 0.05) than those induced by the avirulent strain W7119 [85]. *S. suis* also induced pyroptosis in macrophages (Figure 2B), characterized by cell swelling and the formation of large bubbles from the plasma membrane, leading to increased expression of cytokines IL-1β and IL-18 [86,87]. Infection with PRRSV, ASFV, and Haemophilus parasuis can also induce macrophage apoptosis [88]. ASFV infection led to macrophage apoptosis, which depends on caspase 3, 7, 9, and 12 [89]. In addition, ASFV can also induce pyroptosis of macrophages [90], and the pS273R protein encoded by it alleviates pyroptosis by inactivating the executive protein GSDMD [91].

Regarding cytokine production, both live and heat-killed *S. suis* stimulate macrophages to release pro-inflammatory mediators. This response is independent of phagocytosis and is driven by bacterial components, including the cell wall, rather than capsular material [92]. However, the presence of CPS regulates the interaction between *S. suis* and TLRs. In the absence of CPS, undiscovered cell wall components induce the production of cytokines and chemokines through TLR2-dependent and independent pathways, while CPS promotes the production of MCP-1 in a MyD88-independent manner. The relative expression of CPS may alter the interaction with the host cells, thereby changing the outcome of the inflammatory response [93]. Both virulent and avirulent *S. suis* strains induce the secretion of pro-inflammatory cytokines [85,94]. The production of these cytokines significantly impacts the inflammatory microenvironment and can exacerbate tissue damage in severe infections. In addition, the avirulent strain W7119 adheres more strongly to macrophages than the virulent strain 700794, potentially due to differences in capsular polysaccharide composition, which may facilitate easier phagocytosis [85].

In vitro, Li and Wang et al. demonstrated that *S. suis* can induce macrophage apoptosis by using live-cell imaging and TUNEL assays. Furthermore, p53- and caspase-dependent signaling pathways are implicated in *S. suis*-induced macrophage apoptosis, with significant increases in AIF and cleaved caspase-3 proteins [85]. Also, *S. suis* has been shown to promote macrophage M1 polarization, prime pyroptosis, and activate classical inflammation-related MAPK and AKT signaling pathways, with upregulated phosphorylation of p38 and ERK and increased AKT protein expression. This leads to the production of pro-inflammatory mediators such as TNF-α, IL-8, IL-1β, IL-6, and IL-18, ultimately contributing to programmed cell death [83]. The above findings contribute to understanding lymphocyte depletion in immune organs under *S. suis* infection.

**Figure 3:**
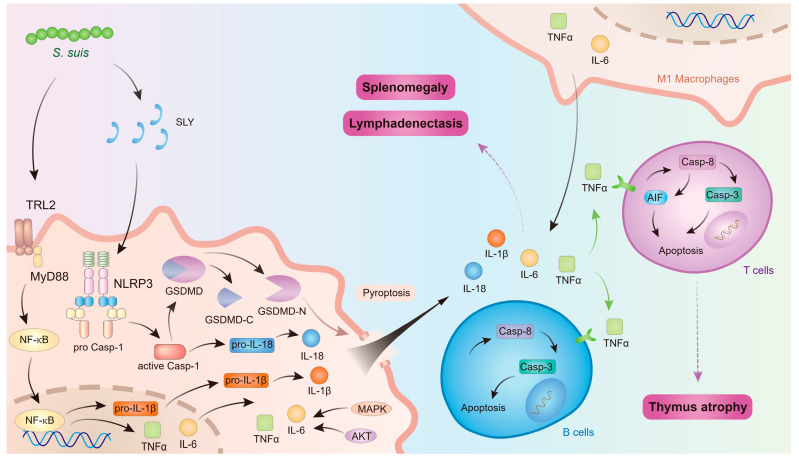
The mechanism of *S. suis* affecting immune organs. The figure was prepared in Adobe Illustrator 2021 v25.0 (Adobe, San Jose, CA, USA).

In conclusion, *S. suis* induces macrophage polarization and pyroptosis in immune organs, thereby releasing a substantial number of inflammatory mediators. These mediators facilitate programmed cell death in immune cells, leading to lymphocyte depletion within immune organs and subsequent thymus atrophy. Simultaneously, lymphocyte depletion in immune organs results in reduced lymphocyte counts in peripheral blood, contributing to immunosuppression. Furthermore, excessive inflammatory mediators cause inflammatory damage to immune organs, manifested as splenomegaly and lymphadenopathy, which further impairs immune organ function and exacerbates systemic immunosuppression (Figure 3).

## 6. Concluding Remarks and Future Prospects

Understanding the mechanisms underlying macrophage polarization and metabolism holds significant therapeutic potential for inflammatory and immune-related diseases. For instance, miRNAs such as miR-155 (M1) and miR-223 (M2) regulate macrophage phenotypes and represent promising therapeutic targets [95]. Modulating macrophage activity may lead to innovative strategies for treating fibrosis and chronic inflammatory conditions [96].

Infection with *S. suis* induces apoptosis in CD3^+^ T cells and B cells, resulting in a marked reduction in lymphocytes in immune organs. Consequently, peripheral T cell populations (CD4^+^CD8^−^ and CD4^−^CD8^+^) are depleted, and antibody production is impaired, exacerbating systemic immunosuppression. This process may be mediated by the virulence factor suilysin of *S. suis* [28]. Suilysin is a critical virulence factor for *S. suis,* as it facilitates the formation of pores in the target cell membrane [49]. Given its pivotal role in *S. suis* pathogenicity, suilysin represents an attractive target for the development of innovative antivirulence drugs.

NLRP3 inflammasome activation plays a critical role in *S. suis*-induced blood–brain barrier disruption and streptococcal toxic shock-like syndrome (STSLS) [50,97]. NLRP3 inflammasome hyperactivation induces NLRP3-GSDMD-dependent pyroptosis and NLRP3-mediated cytokine storm, leading to the substantial production of apoptosis-related cytokines such as TNF-α [47,86]. Since NLRP3 and TNF-α are key factors contributing to apoptosis and tissue damage, the development of novel biological agents targeting NLRP3 and TNF-α to alleviate immune organ damage caused by *S. suis* infection is considered essential.
